# White matter microstructural integrity as a key to effective propagation of gamma entrainment in humans

**DOI:** 10.1007/s11357-024-01281-2

**Published:** 2024-07-15

**Authors:** Yeseung Park, Euisuk Yoon, Jieun Park, Jun Sung Kim, Ji Won Han, Jong Bin Bae, Sang-Su Kim, Do-Won Kim, Se Joon Woo, Jaehyeok Park, Wheesung Lee, Seunghyup Yoo, Ki Woong Kim

**Affiliations:** 1 Department of Neuropsychiatry, Seoul National University Bundang Hospital, Seongnam, Korea; 2 Department of Brain and Cognitive Science, College of Natural Sciences, Seoul National University, Seoul, Korea; 3 Department of Biomedical Engineering, Chonnam National University, Yeosu, Republic of Korea; 4 Department of Ophthalmology, College of Medicine, Seoul National University, Seoul, Republic of Korea; 5 Department of Ophthalmology, Seoul National University Bundang Hospital, Seongnam, Republic of Korea; 6 School of Electrical Engineering, Korea Advanced Institute of Science and Technology (KAIST), Daejeon, Republic of Korea; 7 Department of Psychiatry, College of Medicine, Seoul National University, Seoul, Korea; 8 Department of Health Science and Technology, Seoul National University Graduate School of Convergence Science and Technology, Suwon, Korea; 9 Department of Neuropsychiatry, College of Medicine, Seoul National University, Seoul, Korea; 10 Seoul National University Bundang Hospital, 82, Gumi-ro 173 beon-gil, Bundang-gu, Seongnam-si, Gyeonggi-do 13620 Republic of Korea

**Keywords:** Gamma rhythm, Photobiomodulation, Alzheimer’s disease, White matter, DTI

## Abstract

Gamma entrainment through sensory stimulation has the potential to reduce the pathology of Alzheimer’s disease in mouse models. However, clinical trials in Alzheimer’s disease (AD) patients have yielded inconsistent results, necessitating further investigation. This single-center pre-post intervention study aims to explore the influence of white matter microstructural integrity on gamma rhythm propagation from the visual cortex to AD-affected regions in 31 cognitively normal volunteers aged ≥ 65. Gamma rhythm propagation induced by optimal FLS was measured. Diffusion tensor imaging was employed to assess the integrity of white matter tracts of interest. After excluding 5 participants with a deficit in steady-state visually evoked potentials, 26 participants were included in the final analysis. In the linear regression analyses, gamma entrainment was identified as a significant predictor of gamma propagation (*p* < 0.001). Furthermore, the study identified white matter microstructural integrity as a significant predictor of gamma propagation by flickering light stimulation (*p* < 0.05), which was specific to tracts that connect occipital and temporal or frontal regions. These findings indicate that, despite robust entrainment of gamma rhythms in the visual cortex, their propagation to other regions may be impaired if the microstructural integrity of the white matter tracts connecting the visual cortex to other areas is compromised. Consequently, our findings have expanded our understanding of the prerequisites for effective gamma entrainment and suggest that future clinical trials utilizing visual stimulation for gamma entrainment should consider white matter tract microstructural integrity for candidate selection and outcome analysis.

**Supplementary information:**

The online version contains supplementary material available at 10.1007/s11357-024-01281-2.

## Introduction

In recent years, there has been growing interest in the potential of modulating brain activity by rhythmic sensory stimulation, in particular gamma rhythms, as a therapeutic approach for Alzheimer’s disease (AD) [1–3]. Gamma rhythms, which are intrinsic oscillatory patterns in the 30–80 Hz frequency range, have been linked to various cognitive processes, including attention [4], memory retention [5], and sensory perception [6]. It has been suggested that alterations in these rhythms may be linked to the cognitive deficits observed in AD, which could make them a focus for therapeutic intervention [1–3]. Furthermore, gamma entrainment using sensory stimulation such as 40 Hz flickering light stimulation (FLS) has been demonstrated to reduce brain beta-amyloid (Aβ) and phosphorylated tau and to improve cognitive function in transgenic AD mice [7–9]. Gamma entrainment is a process whereby brain activity is synchronized to external stimuli at gamma frequencies [10].

While encouraging findings have been observed in preclinical studies [7–9], the clinical trials investigating the efficacy of gamma entrainment using 40 Hz combined visual and auditory stimulation in patients with AD have yielded inconsistent results [11–13]. Our previous work indicated that the discrepancy in the efficacy of gamma entrainment using sensory stimulation between mice and humans may be attributable to the potential differences in the optimal parameters of sensory stimulation for entraining gamma rhythms between mice and humans. For example, 32 Hz was more effective for gamma entrainment using FLS than 40 Hz in older adults in our previous work. The 32 Hz FLS entrained gamma rhythms 200% more strongly and 125% more widely in 137% more participants than the 40 Hz FLS [14]. In addition, the intensity of visual stimulation used in previous clinical trials varied considerably, ranging widely from 192 to 1400 cd/m^2^ [11–13, 15, 16]. In our previous work in diurnal humans, we found that 700 cd/m^2^ FLS entrained gamma rhythms in the visual cortex 107% and 156% more than 400 cd/m^2^ and 100 cd/m^2^, respectively, and propagated gamma rhythms 125% more to frontotemporal regions than 400 cd/m^2^ FLS [14]. These results suggest that gamma entrainment and propagation may be considerably influenced by the light intensity of visual stimulation. Previous preclinical studies employed rather strong light stimulation for nocturnal mice. They used approximately 300 cd/m^2^ FLS, which is double the 150 cd/m^2^ that has been shown to induce retinal neurodegeneration in nocturnal mice [17, 18].

However, it is important to note that the lack of consistent observations of gamma entrainment in clinical trials cannot be attributed solely to inter-species differences in extrinsic factors such as frequency or intensity of external sensory stimulation. It is also due to inter-individual differences in the intrinsic factors involved in the spread of gamma rhythms in humans. It is clear that if the gamma rhythms entrained in the sensory cortex by external sensory stimuli are not properly propagated to other brain regions where the pathology of AD occurs, gamma entrainment using sensory stimulation may not have the desired therapeutic effect in patients with AD. Propagation refers to the spread of gamma rhythms, which are initially entrained in the sensory cortex synchronized to external stimuli, to other interconnected brain regions, facilitating coordinated neural activity and information processing [19]. In propagating gamma rhythms entrained in the primary sensory cortex to other brain regions affected by AD including the temporal and frontal regions [20–25], the microstructural integrity of white matter plays a pivotal role. However, the microstructural integrity of white matter differs considerably not only between patients with AD [26] but also between normal older adults without AD [27–29].

This single-center, pre-post-intervention study aimed to determine whether the microstructural integrity of white matter tracts influences the spread of gamma rhythms induced by optimal FLS in cognitively normal older adults. It was hypothesized that the spread of gamma rhythms entrained in the sensory cortex to other brain regions will decrease as the white matter microstructural integrity connecting them decreases. By doing so, we sought to gain a clearer understanding of why gamma entrainment may benefit some individuals but not others, potentially leading to more targeted and effective therapeutics.

## Materials and methods

### Participants

We enrolled 31 cognitively normal volunteers (16 men and 15 women) aged 69.8 ± 2.3 years (65–76 years). Geriatric psychiatrists evaluated each participant with a standardized face-to-face diagnostic interview, physical and neurological examinations, and laboratory tests using the Korean version of the Consortium to Establish a Registry for Alzheimer’s Disease (CERAD-K) Clinical Assessment Battery [30] and the Korean version of the Mini International Neuropsychiatric Interview [31]. Neuropsychologists or trained research nurses assessed cognitive function using the CERAD-K Neuropsychological Assessment Battery, Digit Span Test, and Frontal Assessment Battery [32–34], and depressive symptoms using the Korean version of the Geriatric Depression Scale (GDS) [35]. The CERAD-K Neuropsychological Assessment Battery consists of the following neuropsychological tests: Verbal Fluency Test, 15-item Boston Naming Test, Mini Mental State Examination (MMSE), Word List Memory Test, Constructional Praxis Test, Word List Recall Test, Word List Recognition Test, Constructional Recall Test, and Trail Making Test A/B. Ophthalmologists also evaluated each participant using the forced visual activity test, slit lamp examination, fundoscopy, and optical coherence tomography.

All participants were living in the community independently, performed all neuropsychological tests above − 1.5 standard deviation of the age-, sex-, and education-adjusted norms of normal elderly Koreans, and had a clinical dementia rating of 0. None had a current or previous history of major psychiatric, neurological, or ophthalmologic disorders. All participants were right-handed and had normal hearing and normal or corrected-to-normal vision.

All participants gave written informed consent to participate in this study. The study protocol was approved by the institutional review board of Seoul National University of Bundang Hospital (B-1809-493-004).

### Gamma entrainment using FLS

This study builds upon our previous work [14]. Our previous study aimed to identify the optimal FLS parameters for entraining gamma rhythms in healthy older adults. In recent clinical trials, combined auditory and visual stimulation was employed for gamma entrainment [8, 12, 13] because it entrained gamma rhythms more widely than visual stimulation alone in preclinical studies. However, in the present study, visual stimulation was employed instead of combined auditory and visual stimulation for the following reasons. Firstly, the objective of this study is not to ascertain which sensory stimuli are more efficacious at entraining gamma rhythms. Rather, it is to investigate the impact of white matter microstructural integrity on the propagation of entrained gamma rhythms. It is more advantageous to test our hypothesis if the region from which gamma rhythms are entrained is one than two in selecting target white matter tracts. Second, it seems clear that visual stimulation plays a pivotal role in gamma entrainment in preclinical studies. This is evidenced by the fact that auditory sensory stimulation alone is insufficient to entrain gamma rhythms [8, 36, 37]. Third, our previous study demonstrated that visual stimulation alone, without combined auditory stimulation, effectively entrained gamma rhythms, including the temporal and frontal regions, when its parameters were optimized for older humans [14, 38]. Fourth, it may be the case that visual stimulation alone is more widely applicable in older adults, in whom hearing difficulty is prevalent, than combined visual and auditory stimulation [39]. Finally, if the effectiveness is the same, it may be the case that using only visual stimulation would be cheaper and easier to use than using combined visual and auditory stimulation.

We conducted resting-state EEG (rsEEG) for 5 min prior to FLS. The FLS was administered in a randomized order with one of the four types (400 cd/m^2^ white light, 700 cd/m^2^ white light, 400 cd/m^2^ red light, and 700 cd/m^2^ red light) in each experimental session. The apparatus consisted of a pair of white organic light-emitting diode panels (4.7 cm × 4.7 cm; color temperature 3000 K; LG Display Co., Ltd., Seoul, Korea) attached to a pair of glasses. The steady-state visually evoked potentials (SSVEP) of gamma rhythms were measured for 4000 ms from 1000 ms before FLS onset to 1000 ms after FLS offset.

Although the precise mechanisms remain unclear, it is evident that higher levels of light intensity lead to stronger gamma entrainment in response to flickering light stimuli. Previous studies have employed light intensities within the range of 130 to 1400 cd/m^2^, with higher intensities demonstrating the capacity to entrain stronger gamma rhythms [11–13, 15, 16]. Our previous studies examined and compared the strength of gamma entrainment and gamma propagation based on light intensity of 10, 100, 400, and 700 cd/m^2^ [14, 38]. The results indicated that the highest light intensity, 700 cd/m^2^, was the most optimal for stronger gamma entrainment and propagation.

Previous studies have employed a single frequency, 40 Hz, for the stimulation of flickering light [11–13]. However, it is possible that the optimal gamma entrainment frequency may differ between humans and mice. Consequently, our previous study investigated gamma entrainment and propagation based on a frequency range of 32 ~ 50 Hz [38]. For older adults, the low gamma frequency of 32 Hz entrained and propagated the gamma frequency the most [14].

The administration of the FLS has demonstrated that among the light colors, blue, green, red, and white, white and red induce gamma brain rhythms the strongest, with white having fewer side effects than red [38]. Consequently, in this study, we utilized SSVEP and sGC of gamma rhythms entrained by a 32 Hz white FLS of 700 cd/m^2^, as this had optimally entrained and propagated gamma rhythms in older adults [14]. Further details of the FLS administration are provided in our previous study [14].

### Recording, preprocessing, and analysis of EEG

EEG was recorded using 64 Ag–AgCl electrodes on elastic caps (Easycap, EASYCAP GmbH, Munich, Germany) according to the extended International 10–20 System. FCz was used as the reference electrode. The forehead was used as the ground electrode and a pair of electrodes was placed above and below the left eye to record the EEG. The electrodes were kept at an impedance ≤ 10 kΩ throughout the recording period. A 24-bit ActiCHamp DC amplifier and BrainVision Recorder (Brain Products GmbH, Gliching, Germany) were used to amplify and store the EEG signal. The sampling rate was 1000 Hz. No online filters were used in the EEG recordings. Stimulus markers were provided by the FLS control system and synchronized with the rsEEG recording.

We preprocessed and analyzed the data using MATLAB (The MathWorks Inc., Natick, MA, USA), EEGLAB [40], and the BSMART [41] toolbox. We filtered the recorded signals using a 1-Hz high-pass finite impulse response filter and a 60-Hz notch filter, and then applied them to a common mean reference. We performed an independent component analysis to remove eye blinks and other ocular artifacts from the EEG signals. After preprocessing, we segmented the 5-min rsEEG recordings into 1500-ms epochs and then randomly selected 20 artifact-free epochs from them. We obtained a 4000-ms epoch from 1000 ms before FLS onset to 1000 ms after FLS offset, resulting in 204,000-ms epochs of optimal FLS. For the sGC analysis, we obtained 1500 ms from 501 to 2000 ms from each 4000-ms epoch.

We defined the signal-to-noise ratio (SNR) as the ratio of the SSVEP power at the stimulus frequency to the mean power of the background noise from 1 to 60 Hz from 501 to 2000 ms after FLS onset in a logarithmic scale [42], and considered data with less than 1.5 SNR at one or more occipital lobe electrodes (POz, Oz, O1, and O2) as SSVEP deficit due to failed gamma training.

We analyzed the FLS-induced EEG spectral power change using event-related spectral perturbation in each block. We calculated the time-frequency spectrum of each epoch and normalized the spectrum by dividing the mean power of the pre-stimulus intervals. We calculated the ratio of the EEG’s spectral power during the FLS to that during the pre-FLS interval of each FLS. We then calculated the event-related desynchronization/synchronization (ERD/ERS) value by averaging the EEG spectral power ratios for a given optimal FLS from 20 FLS trials to obtain a normalized, averaged FLS-induced spectral power change. We used the ERS to measure the strength of FLS-entrained gamma rhythms and used the average ERS from a 501-ms stimulus onset to a 2000-ms stimulus onset, which was when a gamma rhythm was continuously and strongly entrained, in the analyses of gamma entrainment.

To investigate whether gamma rhythms entrained in the visual cortex propagate to other brain regions, we compared the sGCs during FLS with those at resting state. We calculated the sGCs for all possible electrode pairs and averaged each FLS frequency for each session. We set the time lags for 75 samples to calculate sGC [43]. We compared the mean sGC of the 20 1500-ms epochs from rsEEG to that of the 20 1500-ms epochs from SSVEP during FLS. Since the EEG of each FLS condition had different pairs of significant connections between electrodes, we used graph theory measures to quantitatively compare the network structures between FLS intensities, colors, and frequencies [44, 45]. We compared the number of edges where the sGCs of occipital to frontal or temporocentral connections significantly increased after FLS compared with rsEEG. We examined the propagation of entrainment with sGC by designating the following regions: occipital (POz, Oz, O1, O2), left temporocentral (FT7, T7, TP7, TP9, FC5, FC3, C5, C3, CP5, CP3), right temporocentral (FT8, T8, TP8, TP10, FC4, FC6, C4, C6, CP4, CP6), left frontal (AF7, AF3, F7, F5, F7, F5, FC5, FC3), right frontal (AF4, AF8, F6, F8, F6, F8, FC4, FC6) [46–48]. Using these regions, we calculated the connectivity from the occipital to the temporocentral or frontal regions. We then selected Oz as a representative channel of the occipital region to investigate the association between the microstructural integrity of white matter tracts and entrainment, as Oz is considered a direct entrainment signal from the occipital region [49, 50].

### Acquisition, preprocessing, and analysis of DTI

Diffusion tensor image (DTI) was performed using a 3.0-Tesla Achieva scanner (Philips Medical Systems; Eindhoven, NL). We used a DTI DwiSE sequence (*echo time* = 49 ms; *repetition time* = 5165 ms; *axial plane acquisition matrix size* = 112 × 112 mm; *slice thickness* = 2 mm; *acquired voxel size* = 2.00 × 2.00 × 2.00 mm; and *flip angle* = 90°) and acquired a baseline image (*b* = 0) and 14 different diffusion orientations with a *b*-value of 1000-s/mm^2^.

We then performed DTI parameter acquisition using the FMRIB Software Library (FSL 6.0, https://fsl.fmrib.ox.ac.uk/fsl/fslwiki/) [51] to capture the microstructural integrity of the white matter tracts of interest. First, we converted DTI Digital Imaging and Communication in Medicine volumes to NIfTI format and extracted the brain using the “bet” function and the baseline image using the “fslroi” function on FSL. We then corrected for inhomogeneity using the eddy current function to remove distortion and motion. We generated DTI metric maps for each subject using the “dtifit” function on FSL: fractional anisotropy (FA), mean diffusivity (MD), radial diffusivity (RD), and axial diffusivity (AxD). FA is calculated from each of the eigenvalues that represent the degree of anisotropy of the water molecules [52, 53]. MD represents the overall degree of diffusion, and is calculated as the average of three eigenvalues [52, 54]. RD represents the degree of water diffusion diffusing perpendicular to the tract, indicated by the average of the two smaller eigenvalues, and AxD represents the average diffusion coefficient of water molecules diffusing parallel to the tract at each voxel, indicated by the largest of the three eigenvalues [55, 56]. We registered each participant’s FA map in the FMRIB58 standard space [57, 58] and used this transformation to register each participant’s DTI maps, defined in this standard space back into each participant’s space.

**Fig. 1:**
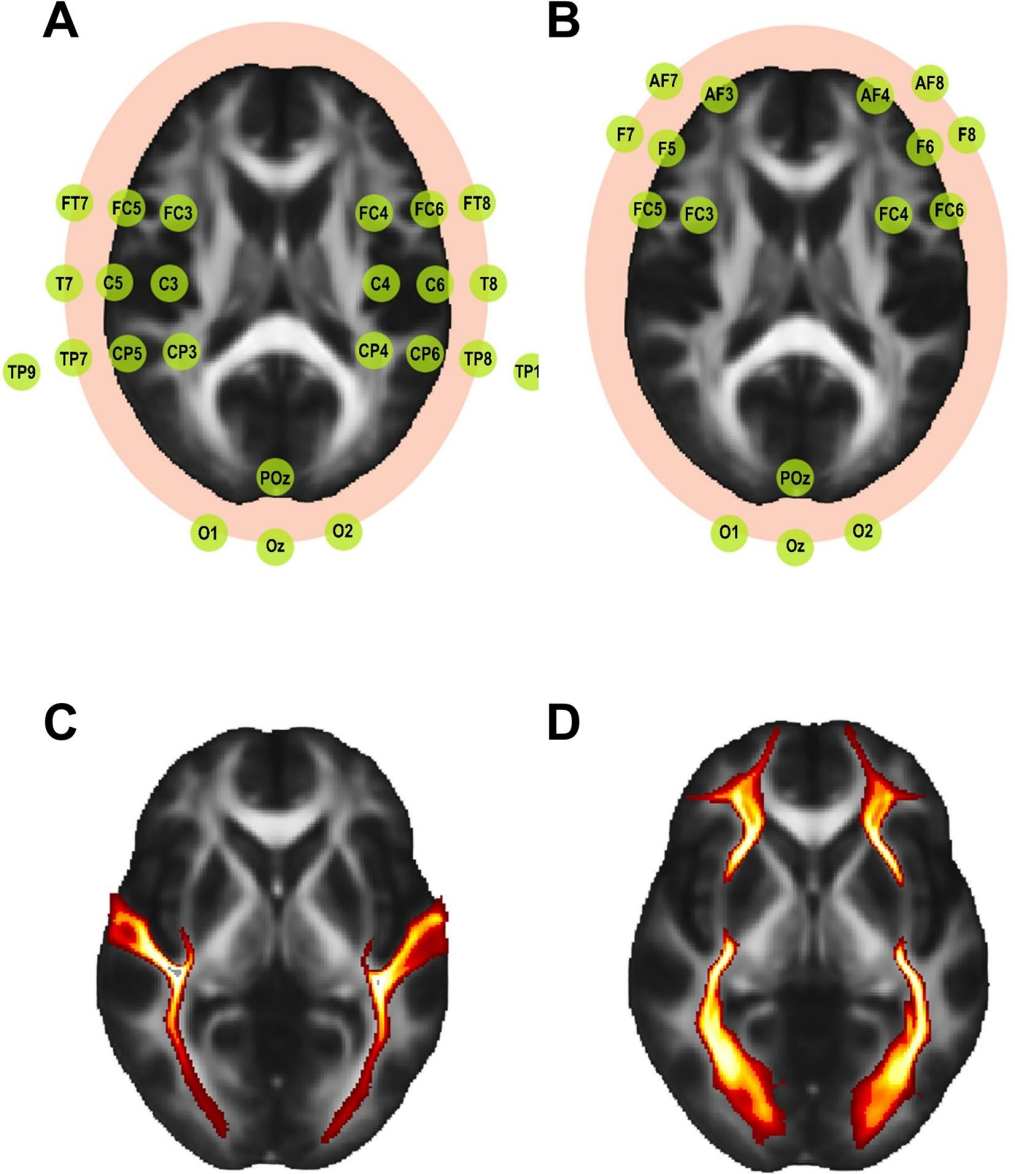
Anatomical location of the tract and electrodes used for the analysis. **A** Brain regions and electroencephalography channels to identify gamma rhythm connectivity from occipital region to temporocentral region in each hemisphere. **B** Brain regions and EEG measurement channels to identify gamma rhythm connectivity from occipital region to frontal region in each hemisphere. **C** Anatomical location of the middle longitudinal fasciculus. **D** Anatomical location of the inferior fronto-occipital fasciculi

Using the FSL atlas tool, we defined four white matter tracts of interest that may play a key role in gamma rhythm entrainment and propagation from occipital to temporal and frontal regions that are known to be affected by AD [59, 60]. We chose “XTRACT HCP tract atlases” as it covers the tracts that we aimed to analyze, and provides robust data quality for human subjects [61]. We defined the left and right middle longitudinal fasciculi (l-MLF and r-MLF), which are distributed from the occipital area to the temporocentral area, and the left and right inferior fronto-occipital fasciculi (l-IFO and r-IFO), which are distributed from the occipital area to the frontal area (Fig. 1). Using the FSL atlas tool, we defined four white matter tracts of interest that may have no association with the gamma rhythm entrainment and propagation from occipital to temporal and frontal regions that are known to be affected by AD. We defined the left and right uncinate fasciculi (l-UF and r-UF), which are distributed from the temporal lobe and frontal lobe, and the left and right vertical occipital fasciculi (l-VOF and r-VOF), which are distributed from the dorsolateral and ventrolateral visual cortex (Supplementary Fig. 1) [61]. To minimize the partial volume effect, we obtained a mean skeleton mask by performing tract-based spatial statistics on the FA maps of all participants using the FSL 6.0 (FMRIB Software Library, https://fsl.fmrib.ox.ac.uk/fsl/fslwiki/) [51]. First, the FA maps of all subjects were registered to the FMRIB58 template and transformed to MNI 152 space. Following this process, a mean FA map was generated, which served as the basis for the construction of the mean skeleton mask. After constructing the mean skeleton mask, we extracted only the overlapping regions by superimposing our extracted ROIs on the mean skeleton mask, with a 20% probability threshold applied to each ROI. We then acquired the mean FA, MD, RD, and AxD values within each ROI.

### Statistical analyses

We compared continuous and categorical variables between groups using the Mann–Whitney *U* test and the Fisher’s exact test, respectively, to test the difference between excluded and included participants.

We conducted a correlation analysis of the ERS of gamma entrainment or spectral Granger causality of gamma connectivity from the occipital to temporocentral and frontal cortices with continuous participant characteristics (age, education, MMSE, and GDS) using the Pearson correlation analysis and with categorical participant characteristics (sex) using point biserial correlation analysis.

We conducted multiple linear regression analysis to examine the potential associations between ERS at Oz (ERS_Oz_) and FA, MD, RD, and AxD of the MLF and IFO on the sGC of gamma rhythm connectivity from the occipital to temporocentral and frontal cortices, respectively. We then proceeded to perform a forward stepwise multiple linear regression analysis to identify significant predictors of the sGC of gamma rhythm connectivity from the occipital to temporocentral and frontal cortices between ERS_Oz_ and FA, MD, RD, and AxD of the MLF and IFO in both hemispheres. We conducted multiple linear regression analysis with ERS_Oz_ and FA as a predictor in Model 1, ERS_Oz_ and MD as a predictor in Model 2, ERS_Oz_ and RD as a predictor in Model 3, and ERS_Oz_ and AxD as a predictor in Model 4.

We conducted forward stepwise multiple linear regression analysis to identify potential predictors of the sGC of gamma rhythm connectivity from the occipital to temporocentral and frontal cortices between ERS_Oz_ and FA, MD, RD, and AxD of the UF and VOF in both hemispheres. We conducted multiple linear regression analysis with ERS_Oz_ and FA as a predictor in Model 1, ERS_Oz_ and MD as a predictor in Model 2, ERS_Oz_ and RD as a predictor in Model 3, and ERS_Oz_ and AxD as a predictor in Model 4.

We used SPSS in Windows (version 20.0; IBM Co., Armonk, NY, USA) and MATLAB (The MathWorks Inc., Natick, MA, USA) for all analyses.

## Results

Of the 31 participants, 26 were included in the final analysis after excluding 5 participants who had an SSVEP deficit. The mean SNR of the excluded participants was below 0 (− 3.33 ± 2.59), indicating that gamma rhythms were not entrained. All the excluded participants were male. Age, education level, MMSE score, GDS score, and FA, MD, RD, and AxD of MLFs and IFOs were comparable between the included and excluded participants (Table 1).

**Table 1:** Characteristics of the participants

	Included (*n* = 26)	Excluded (*n* = 5)	p^*^
Age, years	69.8 (2.8)	69.6 (3.0)	0.856
Women	15 (57.7)	0 (0)	0.043
Education, years	11.7 (4.4)	11.4 (6.0)	0.775
MMSE, points	28.2 (2.0)	28.4 (0.9)	0.696
GDS, points	8.1 (5.1)	4.6 (4.8)	0.195
SNR^a^	5.471 (2.851)	− 3.412 (2.553)	< 0.001
Fractional anisotropy			
Left MLF	0.477 (0.024)	0.472 (0.031)	0.815
Right MLF	0.484 (0.049)	0.480 (0.035)	0.584
Left IFO	0.487 (0.027)	0.487 (0.032)	0.856
Right IFO	0.478 (0.039)	0.481 (0.040)	0.897
Mean diffusivity, 10^−3^ mm^2^/s			
Left MLF	0.958 (0.206)	1.051 (0.298)	0.235
Right MLF	0.952 (0.217)	0.981 (0.153)	0.129
Left IFO	0.934 (0.190)	0.962 (0.153)	0.176
Right IFO	0.930 (0.213)	0.921 (0.106)	0.235
Radial diffusivity, 10^−3^ mm^2^/s			
Left MLF	0.678 (0.130)	0.779 (0.246)	0.129
Right MLF	0.682 (0.189)	0.728 (0.150)	0.159
Left IFO	0.661 (0.136)	0.712 (0.162)	0.195
Right IFO	0.672 (0.174)	0.702 (0.157)	0.417
Axial diffusivity, 10^−3^ mm^2^/s			
Left MLF	1.481 (0.236)	1.554 (0.311)	0.358
Right MLF	1.467 (0.219)	1.473 (0.136)	0.280
Left IFO	1.449 (0.195)	1.439 (0.094)	0.448
Right IFO	1.422 (0.208)	1.391 (0.081)	0.658

*GDS* Geriatric Depression Scale, *MLF* middle longitudinal fasciculus, *MMSE* Mini Mental Status Exam, *IFO* inferior fronto-occipital fasciculus, *SNR* signal-to-noise ratio

Note. Continuous variables are presented as the mean (standard deviation) and categorical variables as numbers (percentages)

^*^Mann–Whitney *U* test for continuous variables and Fisher’s exact test for categorical variables

^a^Ratio of the power of steady-state visually evoked potential at the frequency of administered flickering light

**Fig. 2:**
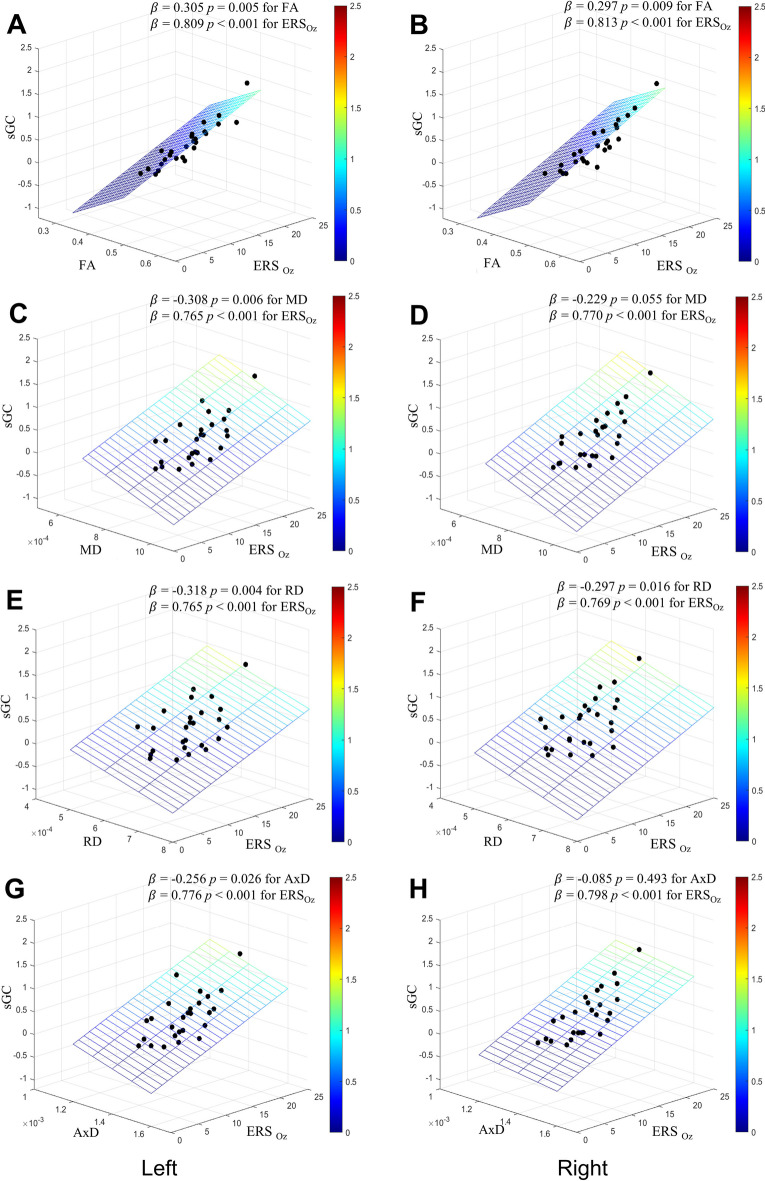
Effects of the strength of gamma entrainment in visual cortex and the microstructural integrity of the middle longitudinal fasciculus on the strength of gamma rhythm connectivity from the occipital region to the temporocentral region^*^. **A** FA of left MLF; **B** FA of right MLF; **C** MD of left MLF; **D** MD of right MLF; **E** RD of left MLF; **F** RD of right MLF; **G** AxD of left MLF; **H** AxD of right MLF. AxD, axial diffusivity; ERS_Oz_, event-related synchronization of the gamma rhythm at Oz of each hemisphere; FA, fractional anisotropy; MD, mean diffusivity; MLF, middle longitudinal fasciculus; RD, radial diffusivity; sGC, spectral Granger causality of gamma rhythm connectivity from occipital region to temporocentral region in each hemisphere. ^*^Multiple linear regression analysis with ERS_Oz_ and one of FA, MD, RD, or AxD of MLF as independent variables and sGC as a dependent variable in each hemisphere. The sGCs of gamma rhythm connectivity from the occipital to the temporocentral regions increased with increasing ERS_Oz_ and FA of MLF while MD, RD, and AxD of MLF decreased in both hemispheres. This suggests gamma rhythm connectivity from the occipital to the temporocentral regions are not only influenced by gamma entrainment, but also influenced by the WM integrity of MLF

**Fig. 3:**
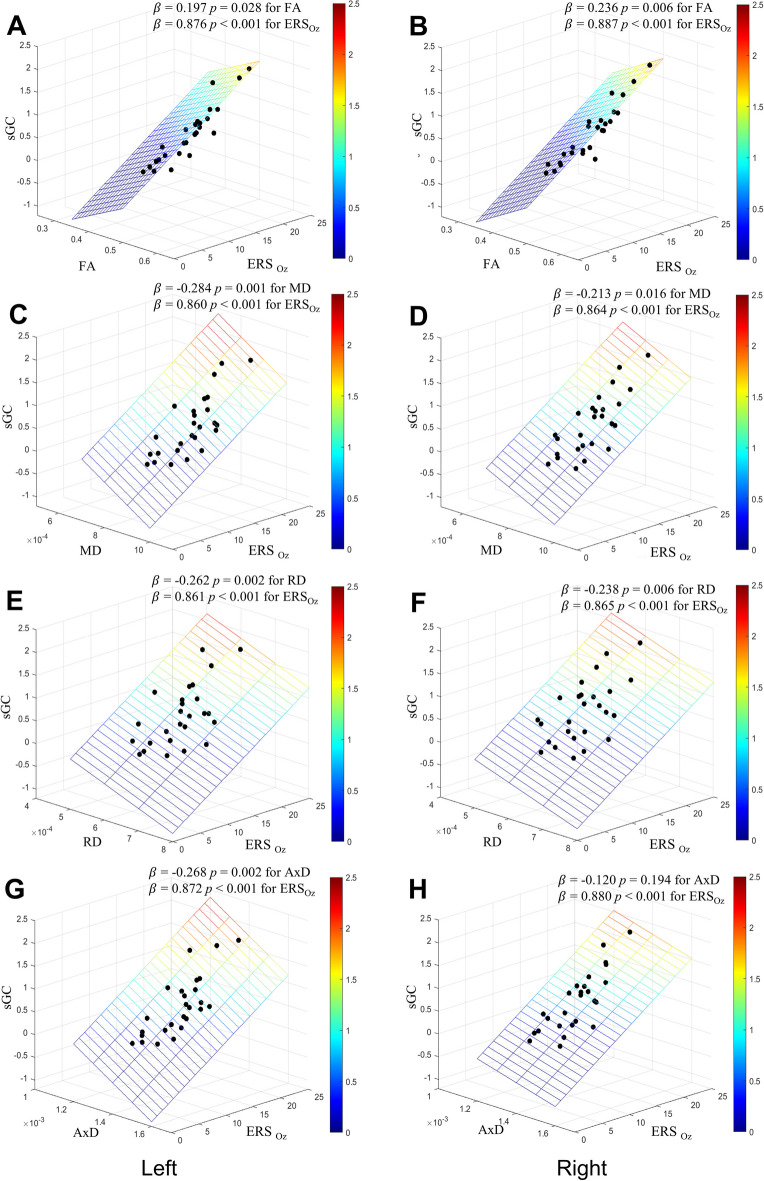
Effects of the strength of gamma entrainment in visual cortex and the microstructural integrity of the inferior fronto-occipital fasciculus on the strength of gamma rhythm connectivity from the occipital region to the frontal region^*^. **A** FA of left IFO; **B** FA of right IFO; **C** MD of left IFO; **D** MD of right IFO; **E** RD of left IFO; **F** RD of right IFO; **G** AxD of left IFO; **H** AxD of right IFO. AxD, axial diffusivity; ERS_Oz_, event-related synchronization of the gamma rhythm at Oz of each hemisphere; FA, fractional anisotropy; MD, mean diffusivity; RD, radial diffusivity; sGC, spectral Granger causality of gamma rhythm connectivity from occipital region to temporocentral region in each hemisphere; IFO, inferior fronto-occipital fasciculi. ^*^Multiple linear regression analysis with ERS_Oz_ and one of FA, MD, RD, or AxD of IFO as independent variables and sGC as a dependent variable in each hemisphere. The sGCs of gamma rhythm connectivity from the occipital to the frontal regions also increased with increasing ERS_Oz_ and FA of IFO while MD, RD, and AxD of IFO decreased in both hemispheres. This suggests gamma rhythm connectivity from the occipital to the frontal regions are not only influenced by gamma entrainment, but also influenced by the WM integrity of IFO

There was no significant correlation between participant characteristics such as age, sex, education, MMSE, and GDS and the ERS_OZ_ or sGCs of gamma rhythm connectivity from the occipital to temporocentral and frontal cortices (*p* > 0.05, Supplementary Table 1). In the multiple linear regression analyses, the sGCs of gamma rhythm connectivity from the occipital to the temporocentral regions increased with increasing ERS_Oz_ and FA of MLF while decreasing MD, RD, and AxD of MLF in both hemispheres (Fig. 2). The sGCs of gamma rhythm connectivity from the occipital to the frontal regions also increased with increasing ERS_Oz_ and FA of IFO while decreasing MD, RD, and AxD of IFO in both hemispheres (Fig. 3).

In the forward stepwise multiple linear regression analyses for MLF and IFO (Table 2), ERS_Oz_ was identified as a significant predictor of sGCs of gamma connectivity from occipital to the temporocentral and the frontal regions in all models (*p* < 0.001), suggesting that the strength of gamma entrainment in visual cortex may be a prerequisite for gamma rhythms to propagate adequately to other brain regions. The study also identified several metrics of WM microstructural integrity (FA, MD, RD, and AxD of MLF and IFO in both hemispheres) as significant predictors of sGCs of gamma connectivity from occipital to the temporocentral and the frontal regions in all models (*p* < 0.05), except AxD of right MLF and IFO and MD of right MLF. This suggests that, despite the robust entrainment of gamma rhythms in the visual cortex, their propagation to other regions may be impaired if the microstructural integrity of the WM tracts connecting the visual cortex to other areas is compromised. The adjusted *R*^2^ value, representing the percentage of fluctuation in the dependent variable that can be explained by the independent variable(s), varied from 0.718 to 0.870 across models. Of these, the adjusted *R*^2^ increased by approximately 4.0 ~ 12.0% across the models (Δ adjusted *R*^2^ = 0.033 ~ 0.091), representing how much the microstructural integrity of the WM has enhanced the fit of the model. This indicates that, regardless of the initial strength of the gamma rhythm entrainment in the visual cortex, WM microstructural integrity plays a significant role in explaining the variation in the strength of gamma connectivity from the occipital region to other parts of the brain.

**Table 2:** Effects of the microstructural integrity of white matter tracts on the spectral Granger causality of gamma connectivity from visual cortex to other brain regions

	Step 1					Step 2					*p for ΔR*^*2*^
	*B*	SE	*Β*	*p*	Adj *R*^2^	*B*	SE	*β*	*p*	Adj *R*^2^	
Left MLF^a^											
Model 1					0.670					0.756	0.005
ERS_Oz_	0.058	0.008	0.826	< 0.001		0.056	0.007	0.809	< 0.001		
FA	-	-	-	-		4.617	1.501	0.305	0.005		
Model 2					0.670					0.754	0.006
ERS_Oz_	0.058	0.008	0.826	< 0.001		0.053	0.007	0.765	< 0.001		
MD	-	-	-	-		− 2048.696	673.895	− 0.308	0.006		
Model 3					0.670					0.761	0.004
ERS_Oz_	0.058	0.008	0.826	< 0.001		0.053	0.007	0.765	< 0.001		
RD	-	-	-	-		− 2253.477	705.365	− 0.318	0.004		
Model 4					0.670					0.724	0.026
ERS_Oz_	0.058	0.008	0.826	< 0.001		0.054	0.007	0.776	< 0.001		
AxD	-	-	-	-		− 1349.610	565.002	− 0.256	0.026		
Right MLF^b^											
Model 1					0.651					0.732	0.009
ERS_Oz_	0.064	0.009	0.816	< 0.001		0.064	0.008	0.813	< 0.001		
FA	-	-	-	-		4.452	1.553	0.297	0.009		
Model 2					0.651						-
ERS_Oz_	0.064	0.009	0.816	< 0.001		-	-	-	-		
MD	-	-	-	-		-	-	-	-		
Model 3					0.651					0.718	0.016
ERS_Oz_	0.064	0.009	0.816	< 0.001		0.060	0.008	0769	< 0.001		
RD	-	-	-	-		− 2510.631	968.499	− 0.279	0.016		
Model 4					0.651						-
ERS_Oz_	0.064	0.009	0.816	< 0.001		-	-	-	-		
AxD	-	-	-	-		-	-	-	-		
Left IFO^c^											
Model 1					0.792					0.825	0.028
ERS_Oz_	0.093	0.009	0.895	< 0.001		0.091	0.009	0.876	< 0.001		
FA	-	-	-	-		3.969	1.691	0.197	0.028		
Model 2					0.792					0.870	0.001
ERS_Oz_	0.093	0.009	0.895	< 0.001		0.090	0.008	0.860	< 0.001		
MD	-	-	-	-		− 3472.756	889.726	− 0.284	0.001		
Model 3					0.792					0.857	0.002
ERS_Oz_	0.093	0.009	0.895	< 0.001		0.090	0.008	0.861	< 0.001		
RD	-	-	-	-		− 3062.857	891.133	− 0.262	0.002		
Model 4					0.792					0.861	0.002
ERS_Oz_	0.093	0.009	0.895	< 0.001		0.091	0.008	0.872	< 0.001		
AxD	-	-	-	-		− 2915.147	814.413	− 0.268	0.002		
Right IFO^d^											
Model 1					0.797					0.848	0.006
ERS_Oz_	0.094	0.009	0.897	< 0.001		0.093	0.008	0.887	< 0.001		
FA	-	-	-	-		4.502	1.490	0.236	0.006		
Model 2					0.797					0.836	0.016
ERS_Oz_	0.094	0.009	0.897	< 0.001		0.091	0.009	0.864	< 0.001		
MD	-	-	-	-		− 2531.416	972.572	− 0.213	0.016		
Model 3					0.797					0.848	0.006
ERS_Oz_	0.094	0.009	0.897	< 0.001		0.091	0.008	0.865	< 0.001		
RD	-	-	-	-		− 2686.499	887.538	− 0.238	0.006		
Model 4					0.797						-
ERS_Oz_	0.094	0.009	0.897	< 0.001		-	-	-	-		
AxD	-	-	-	-		-	-	-	-		

*AxD* axial diffusivity, *ERS*_*Oz*_ event-related synchronization of gamma rhythms at Oz, *FA* fractional anisotropy, *IFO* inferior fronto-occipital fasciculus, *MD* mean diffusivity, *MLF* middle longitudinal fasciculus, *SE* standard error, *RD* radial diffusivity, *ΔR*^*2*^ change in the *R*^2^ between steps

^a^Forward stepwise multiple linear regression analyses computing the spectral Granger causality of gamma rhythm connectivity from left occipital to left temporocentral region as a dependent variable and ERS_Oz_ and one of FA, MD, RD, or AxD of left MLF as independent variables

^b^Forward stepwise multiple linear regression analyses computing the spectral Granger causality of gamma rhythm connectivity from right occipital to right temporocentral region as a dependent variable and ERS_Oz_ and one of FA, MD, RD, or AxD of right MLF as independent variables

^c^Forward stepwise multiple linear regression analyses computing the spectral Granger causality of gamma rhythm connectivity from left occipital to left frontal region as a dependent variable and ERS_Oz_ and one of FA, MD, RD, or AxD of left IFO as independent variables

^d^Forward stepwise multiple linear regression analyses computing the spectral Granger causality of gamma rhythm connectivity from right occipital to right frontal region as a dependent variable and ERS_Oz_ and one of FA, MD, RD, or AxD of right IFO as independent variables

In the forward stepwise multiple linear regression analyses for UF and VOF (Supplementary Tables 2 and 3), ERS_Oz_ was identified as a significant predictor of sGCs of gamma connectivity from occipital to the temporocentral and the frontal regions in all models (*p* < 0.001). However, the study was not able to identify WM microstructural integrity (FA, MD, RD, and AxD of UF and VOF in both hemispheres) as predictors of sGCs of gamma connectivity from occipital to the temporocentral and the frontal regions in all models (*p* > 0.05). This indicates that the gamma propagation to other regions is not influenced by the WM microstructural integrity of UF and VOF but is specific to the WM microstructural integrity of MLF and IFO.

## Discussion

This study reveals that even if gamma rhythms are successfully entrained in the visual cortex with optimal conditions measured by previous studies, which are found to be white-colored 700 cd/m^2^ with 32 Hz, these rhythms may not adequately propagate to other target regions if the microstructural integrity of the WM tracts connecting the visual cortex to these regions is not intact. This was found to be the case for both MLF and IFO in both hemispheres. However, given that damage to WM microstructural integrity is common in older adults [27–29] and in patients with Alzheimer’s disease [26], WM microstructural integrity should be considered as a potential criterion in identifying appropriate candidates for the administration of gamma entrainment as a therapeutic or preventive intervention against Alzheimer’s disease.

In previous research, a decrease in the powers of resting and evoked beta and gamma waves was found during the early stages of AD [62–70]. Notably, in AD-modeled mice, entrained gamma waves demonstrated the ability to clear AD pathologies and improve cognitive function, whereas entrained alpha, beta, and high gamma waves did not significantly influence amyloid beta accumulation or cognitive function [7, 71–73]. Moreover, alpha and beta waves, when entrained, were not effectively propagated to other target brain regions [74–76]. While the correlation between resting gamma power/synchrony in healthy older adults and cognitive tests remains uncertain, in AD patients, gamma wave power exhibited strong correlations with the level of AD pathologies and cognitive test scores [77–80]. Consistent with these findings, the Mini Mental State Examination (MMSE) score showed an association with the relative power of resting gamma waves but not with other frequency bands. This suggests that the decline in resting gamma power may contribute to decreased cognitive function in AD, and restoring gamma waves could potentially improve cognitive function by directly clearing AD pathologies. Resting low gamma band power in the frontal to parietal region appears to be particularly associated with higher-order cognitive functions [81–84]. Interestingly, in cognitively normal older adults with amyloid beta levels below a certain threshold, the relative power of resting gamma waves increases with advancing age, showing a negative correlation with the MMSE score in the current study. However, in AD patients, the power and/or synchrony of gamma waves in response to external stimuli decreases with increasing amyloid beta deposition [2, 3, 13, 77, 78, 85, 86]. These findings show the importance of gamma wave entrainment as a potential therapeutic intervention for AD, given its efficacy in clearing AD pathologies and its correlation with cognitive function, distinct from other frequency bands.

Both the propagation and the resting coherence of gamma rhythms have been observed to be negatively affected by the WM microstructural integrity [21–25, 66]. The microstructural integrity of frontal, temporal, parietal, and occipital white matter not only shows age-related deterioration [27–29] but is also frequently compromised in Alzheimer’s disease patients [26–29]. This deterioration is often accompanied by a decrease in EEG power or synchronization [21, 22, 24]. However, none of the previous preliminary studies investigating the effect of gamma entrainment in patients with Alzheimer’s disease considered WM microstructural integrity in participant selection or outcome analysis [11–13]. Therefore, to underline the critical role of WM microstructural integrity in gamma entrainment using FLS, this study is the first to directly demonstrate the effect of WM microstructural integrity on gamma rhythm propagation. Although WM microstructural integrity accounted for 4.0 ~ 12.0% of the total variances in the strength of gamma rhythm connectivity in healthy younger older adults participating in the current study, its impact may be greater in the patients with Alzheimer’s disease who have more severe impairments in WM microstructural integrity than healthy older adults.

We used four distinct indicators of WM microstructural integrity to delineate structural integrity and investigate its specific structural significance in gamma rhythm propagation. Typically, FA is considered to represent overall neuronal microstructural integrity, MD to be indicative of membrane density, RD to reflect axon myelination, and AxD to indicate axonal injury [87]. Each of these individual WM microstructural integrity indicators showed a significant association with the strength of gamma rhythm connectivity represented by sGC. This aligns with previous studies, which also showed an association between poor microstructural integrity and diminished gamma rhythm functional connectivity [88, 89]. As such, all the WM microstructural integrity metrics we investigated could potentially be used to screen suitable candidates for gamma entrainment using FLS as a therapeutic or preventive intervention.

Of all the WM microstructural integrity metrics, RD was explained in all the multiple linear regression models, indicating a significant association with sGC in all the WM tracts. In contrast, AxD had the fewest number of models to explain a significant association with sGC of WM tracts examined. It is possible that the difference in the number of explained models may be due to the characteristics of our participants. The relationship between AxD and brain health has been a topic of debate, with some studies suggesting a link to [90–92]. Additionally, AxD has been shown to be affected primarily in the later stages of Alzheimer’s disease (AD) [93]. Given that our participants were cognitively healthy, this may have contributed to the limited number of explanatory models for AxD. On the other hand, RD, which is potentially linked to axonal myelination [94, 95], may be a more accurate indicator of white matter integrity for gamma rhythm propagation. This may have reflected the underlying mechanism of propagation. Some studies have postulated that differences in axonal myelination can modulate the speed of signal transmission, allowing synaptic signals to sum even at different distances between distant neurons [96, 97]. It is postulated that more distant neurons may have increased myelination for faster conduction speed. Conversely, neurons in closer proximity may exhibit less myelination to match the conduction speed of neurons in more distant locations [98, 99]. This suggests that the propagation of signals between brain regions may be more closely associated with axonal myelination, and consequently, with RD. It is also worth noting that FA and MD (representing structural integrity and membrane density, respectively) are related to axonal myelination, which may help to explain their strong association with the strength of gamma rhythm connectivity. While the MD for the right MLF model was not significant on sGC, it did show a marginal significance (*p* = 0.055). It is also interesting to note that the model for the right MLF and IFO seems to also have fewer explained models than the left models. This could be attributed to the fact that the right MLF and IFO are denser than the left MLF and IFO [100]. Given that our participants are cognitively normal, it is possible that their MLF and IFO have not deteriorated to the extent that they affect the propagation of gamma rhythms. This could have resulted in a reduction in the statistical power for the model, which suggests that there may be a threshold for WM microstructural integrity to affect gamma propagation. We also do recognize that there may be numerous unknown variables that could have influenced the statistical result. To accurately examine the association between specific metrics of WM microstructural integrity and gamma rhythm propagation, further studies are needed that differentiate between these metrics and include more participants who are very old or have Alzheimer’s disease. Despite these considerations, our study was successful in demonstrating the influence of WM microstructural integrity on gamma rhythm propagation. Therefore, our study suggests that WM microstructural integrity may have potential for optimizing FLS parameters for older adults and for selecting appropriate candidates who may benefit from gamma entrainment using FLS.

The frontal and temporal regions, known to be affected by Alzheimer’s disease, are interconnected by long-distance gamma rhythms [101], necessitating the selection of white matter tracts forming these connections from the occipital region to illustrate the effect of WM microstructural integrity on gamma rhythm propagation. Specifically, MLFs extend from the occipital to the temporocentral region [102] and IFOs project from the occipital to the frontal region [103]. These tracts potentially influence the propagation of gamma rhythms entrained in the visual cortex to the temporocentral and frontal regions [101]. On the contrary, while the WM microstructural integrity UF and VOF is expected to affect EEG as cross-fissural fascicles [104], they do not connect the occipital with temporal or frontal regions. The result of the linear regression model has shown that WM microstructural integrity of MLF and IFO was a significant predictor while that of UF and VOF was not a predictor for the gamma propagation between occipital and temporal or frontal regions. These results clearly demonstrated that the WM microstructural integrity of MLFs and IFOs was significantly associated with the strength of gamma rhythm connectivity between occipital and corresponding target brain regions. It seems plausible to suggest that the microstructural integrity of MLFs and IFOs may be beneficial for enabling the proper propagation of gamma rhythms entrained in the visual cortex to target brain areas in AD patients.

Up to 35% of normal adults aged 18–55 years exhibit an SSVEP deficit within the gamma range [105]. In this study, five out of 31 participants (16.1%) were excluded from the analyses due to SSVEP deficit. Men tend to exhibit SSVEP deficit more than women [105, 106], and in this study, all five excluded participants were men. Although impairment in WM microstructural integrity may increase the risk of SSVEP deficit [106], the values representing WM microstructural integrity were comparable between the included and the excluded participants across all white matter tracts in this study.

There is alternative sensory stimulation to entrain gamma rhythms such as an auditory stimulation or the combination with visual stimulation [8, 12, 13]. However, auditory stimulation has shown limited efficacy in clearing AD pathology beyond the auditory cortex [8, 36, 37]. In contrast, visual stimulation has demonstrated effectiveness in clearing Alzheimer’s pathology not only in the visual cortex but also in the frontal and temporal regions [9, 72]. Moreover, gamma waves induced by visual stimuli exhibit a longer duration compared to those induced by auditory stimuli [107]. Additionally, the prevalence of senile deafness rises in adults over 70 years old [39], while central auditory processing disorders, affecting complex sound recognition, are more common in individuals with MCI [39, 108, 109]. For multimodal stimulation, each modality could desynchronize the gamma entrainment of other modalities [60], which could lessen the efficacy of overall gamma entrainment. Consequently, visual stimuli have proven to be more effective and widely applicable for entraining gamma waves in older adults, particularly those with Alzheimer’s disease.

This study has several limitations. First, surface EEG changes may not accurately reflect those in the source signal due to volume conduction. Therefore, sGC differences between surface EEG electrodes may not be solely attributable to differences in the microstructural integrity of the corresponding WM tracts. Second, this study had limited statistical power because of the small sample size and the modest inter-individual differences in WM microstructural integrity in healthy older adults. Third, the observed relationships between the metrics of WM microstructural integrity and the sGC of gamma rhythm connectivity are correlational and not necessarily causal. Further research may be needed to determine the precise mechanisms by which WM microstructural integrity influences the propagation of gamma rhythms in the brain.

Our study highlights the importance of considering the microstructural integrity of key white matter tracts when assessing the efficacy of gamma entrainment in Alzheimer’s disease patients in future clinical trials. While it remains critical to use an optimized FLS that can strongly entrain gamma rhythms in the visual cortex, the state of MW microstructural integrity should be considered in participant selection and outcome analysis. For individuals with severe impairments in WM microstructural integrity, direct gamma rhythm entrainment in target brain regions using other neuromodulation devices, such as transcutaneous electrical stimulation, may be considered. These findings expand our understanding of the requirements for effective gamma entrainment and have implications for the development of more effective therapeutic or preventive interventions for Alzheimer’s disease.

## Supplementary information

Below is the link to the electronic supplementary material.

Supplementary file1 (DOCX 163 KB)

## Data Availability

The data that support the findings of this study are available on request from the corresponding author.

## Abbreviations

AxD: Axial diffusivity
Aβ: Amyloid beta
CERAD-K: Korean version of the Consortium to Establish a Registry for Alzheimer’s Disease
DTI: Diffusion tensor image
ERD: Event-related desynchronization
ERS: Event-related synchronization
ERSP: Event-related spectral perturbation
FA: Fractional anisotropy
FLS: Flickering light stimulation
GDS: Geriatric Depression Scale
MD: Mean diffusivity
MLF: Middle longitudinal fasciculus
MMSE: Mini Mental Status Exam
MRI: Magnetic resonance image
RD: Radial diffusivity
ROI: Regions of interest
rsEEG: Resting state electroencephalography
SF: Spatial frequency
sGC: Spectral Granger causality
SNR: Signal-to-noise ratio
SSVEP: Steady-state visually evoked potentials
WM: White matter
IFO: Inferior fronto-occipital fasciculi
