# Supplementary material for: White matter microstructural integrity as a key to effective propagation of gamma entrainment in humans
Source: GeroScience. 2024 Jul 15;47(1):1019–37. doi: 10.1007/s11357-024-01281-2 (PMC11872816; doi:10.1007/s11357-024-01281-2)
Supplement: Supplementary file 1 — Supplementary file1 (DOCX 163 KB) [file 11357_2024_1281_MOESM1_ESM.docx]

**Supplementary table 1. Correlation between the participant’s characteristics and the ERS of gamma entrainment or spectral Granger causality of gamma connectivity from visual cortex to other brain regions.**

|  | ERS_Oz_ |  | sGC_l-OTC_ | | sGC_r-OTC_ | | sGC_l-OF_ | | sGC_r-OF_ |  |
| --- | --- | --- | --- | --- | --- | --- | --- | --- | --- | --- |
|  | *r* | *p*^*^ | *r* | *p*^*^ | *r* | *p*^*^ | *r* | *p*^*^ | *r* | *p*^*^ |
| Age, years | -0.009 | 0.966 | 0.122 | 0.584 | 0.024 | 0.910 | -0.035 | 0.867 | -0.027 | 0.897 |
| Sex, women | 0.363 | 0.068 | 0.209 | 0.305 | 0.122 | 0.553 | 0.278 | 0.170 | 0.183 | 0.370 |
| Education, years | -0.281 | 0.164 | -0.083 | 0.696 | -0.085 | 0.682 | -0.082 | 0.699 | -0.061 | 0.771 |
| MMSE, points | -0.150 | 0.463 | 0.108 | 0.599 | 0.106 | 0.608 | 0.053 | 0.799 | 0.007 | 0.975 |
| GDS, points | -0.240 | 0.238 | -0.321 | 0.117 | -0.353 | 0.079 | -0.378 | 0.058 | -0.373 | 0.060 |

ERS_Oz_=event-related synchronization of gamma-rhythms at Oz; GDS= Geriatric Depression Scale; MMSE= Mini Mental Status Exam; sGC_l-OTC_=spectral Granger causality of gamma connectivity from visual cortex to left temporocentral regions; sGC_r-OTC_=spectral Granger causality of gamma connectivity from visual cortex to right temporocentral regions; sGC_l-OF_=spectral Granger causality of gamma connectivity from visual cortex to left frontal regions; sGC_r-OF_=spectral Granger causality of gamma connectivity from visual cortex to right frontal regions;

^*^ Pearson correlation analysis for correlations between continuous variables and point biserial correlation analysis for correlations between continuous and categorical variables.

**Supplementary table 2. Effects of the microstructural integrity of white-matter tracts of no-related tracts on the spectral Granger causality of gamma connectivity from visual cortex to temporocentral regions**

|  | Step 1 |  |  |  |  |  | Rejected variable | |
| --- | --- | --- | --- | --- | --- | --- | --- | --- |
|  | B | SE | β | *p* | Adj R^2^ |  | β | *p* |
| Left UF^a^ |  |  |  |  |  |  |  |  |
| Model 1 |  |  |  |  | 0.670 |  |  |  |
| ERS_Oz_ | 0.058 | 0.008 | 0.826 | <0.001 |  |  |  |  |
| FA | - | - | - | - |  |  | 0.177 | 0.135 |
| Model 2 |  |  |  |  | 0.670 |  |  |  |
| ERS_Oz_ | 0.058 | 0.008 | 0.826 | <0.001 |  |  |  |  |
| MD | - | - | - | - |  |  | -0.062 | 0.617 |
| Model 3 |  |  |  |  | 0.670 |  |  |  |
| ERS_Oz_ | 0.058 | 0.008 | 0.826 | <0.001 |  |  |  |  |
| RD | - | - | - | - |  |  | -0.108 | 0.367 |
| Model 4 |  |  |  |  | 0.670 |  |  |  |
| ERS_Oz_ | 0.058 | 0.008 | 0.826 | <0.001 |  |  |  |  |
| AxD | - | - | - | - |  |  | 0.016 | 0.894 |
| Right UF^b^ |  |  |  |  |  |  |  |  |
| Model 1 |  |  |  |  | 0.651 |  |  |  |
| ERS_Oz_ | 0.064 | 0.009 | 0.816 | <0.001 |  |  |  |  |
| FA | - | - | - | - |  |  | 0.154 | 0.216 |
| Model 2 |  |  |  |  | 0.651 |  |  |  |
| ERS_Oz_ | 0.064 | 0.009 | 0.816 | <0.001 |  |  |  |  |
| MD | - | - | - | - |  |  | -0.059 | 0.634 |
| Model 3 |  |  |  |  | 0.651 |  |  |  |
| ERS_Oz_ | 0.064 | 0.009 | 0.816 | <0.001 |  |  |  |  |
| RD | - | - | - | - |  |  | -0.115 | 0.349 |
| Model 4 |  |  |  |  | 0.651 |  |  |  |
| ERS_Oz_ | 0.064 | 0.009 | 0.816 | <0.001 |  |  |  |  |
| AxD | - | - | - | - |  |  | -0.005 | 0.965 |
| Left VOF^c^ |  |  |  |  |  |  |  |  |
| Model 1 |  |  |  |  | 0. 670 |  |  |  |
| ERS_Oz_ | 0.058 | 0.008 | 0.826 | <0.001 |  |  |  |  |
| FA | - | - | - | - |  |  | -0.044 | 0.711 |
| Model 2 |  |  |  |  | 0. 670 |  |  |  |
| ERS_Oz_ | 0.058 | 0.008 | 0.826 | <0.001 |  |  |  |  |
| MD | - | - | - | - |  |  | 0.021 | 0.860 |
| Model 3 |  |  |  |  | 0. 670 |  |  |  |
| ERS_Oz_ | 0.058 | 0.008 | 0.826 | <0.001 |  |  |  |  |
| RD | - | - | - | - |  |  | -0.061 | 0.604 |
| Model 4 |  |  |  |  | 0. 670 |  |  |  |
| ERS_Oz_ | 0.058 | 0.008 | 0.826 | <0.001 |  |  |  |  |
| AxD | - | - | - | - |  |  | -0.110 | 0.356 |
| Right VOF^d^ |  |  |  |  |  |  |  |  |
| Model 1 |  |  |  |  | 0.651 |  |  |  |
| ERS_Oz_ | 0.064 | 0.009 | 0.816 | <0.001 |  |  |  |  |
| FA | - | - | - | - |  |  | -0.034 | 0.782 |
| Model 2 |  |  |  |  | 0.651 |  |  |  |
| ERS_Oz_ | 0.064 | 0.009 | 0.816 | <0.001 |  |  |  |  |
| MD | - | - | - | - |  |  | -0.213 | 0.405 |
| Model 3 |  |  |  |  | 0.651 |  |  |  |
| ERS_Oz_ | 0.064 | 0.009 | 0.816 | <0.001 |  |  |  |  |
| RD | - | - | - | - |  |  | -0.161 | 0.179 |
| Model 4 |  |  |  |  | 0.651 |  |  |  |
| ERS_Oz_ | 0.064 | 0.009 | 0.816 | <0.001 |  |  |  |  |
| AxD | - | - | - | - |  |  | -0.118 | 0.343 |

AxD=axial diffusivity; ERS_Oz_=event-related synchronization of gamma-rhythms at Oz; FA=fractional anisotropy; MD=mean diffusivity; SE=standard error; RD=radial diffusivity; UF= uncinate fasciculus; VOF=vertical occipital fasciculus;

^a^Forward stepwise multiple linear regression analyses computing the spectral Granger causality of gamma-rhythm connectivity from left occipital to left temporocentral region as a dependent variable and ERS_Oz_ and one of FA, MD, RD or AxD of left UF as independent variables

^b^Forward stepwise multiple linear regression analyses computing the spectral Granger causality of gamma-rhythm connectivity from right occipital to right temporocentral region as a dependent variable and ERS_Oz_ and one of FA, MD, RD or AxD of right UF as independent variables

^c^Forward stepwise multiple linear regression analyses computing the spectral Granger causality of gamma-rhythm connectivity from left occipital to left temporocentral region as a dependent variable and ERS_Oz_ and one of FA, MD, RD or Ax) of left VOF as independent variables

^d^Forward stepwise multiple linear regression analyses computing the spectral Granger causality of gamma-rhythm connectivity from right occipital to right temporocentral region as a dependent variable and ERS_Oz_ and one of FA, MD, RD or AxD of right VOF as independent variables

**Supplementary table 3. Effects of the microstructural integrity of white-matter tracts of no-related tracts on the spectral Granger causality of gamma connectivity from visual cortex to frontal regions**

|  | Step 1 |  |  |  |  |  | Rejected variable | |
| --- | --- | --- | --- | --- | --- | --- | --- | --- |
|  | B | SE | β | *p* | Adj R^2^ |  | β | *p* |
| Left UF^a^ |  |  |  |  |  |  |  |  |
| Model 1 |  |  |  |  | 0.792 |  |  |  |
| ERS_Oz_ | 0.093 | 0.009 | 0.895 | <0.001 |  |  |  |  |
| FA | - | - | - | - |  |  | 0.157 | 0.092 |
| Model 2 |  |  |  |  | 0.792 |  |  |  |
| ERS_Oz_ | 0.093 | 0.009 | 0.895 | <0.001 |  |  |  |  |
| MD | - | - | - | - |  |  | -0.097 | 0.320 |
| Model 3 |  |  |  |  | 0.792 |  |  |  |
| ERS_Oz_ | 0.093 | 0.009 | 0.895 | <0.001 |  |  |  |  |
| RD | - | - | - | - |  |  | -0.117 | 0.216 |
| Model 4 |  |  |  |  | 0.792 |  |  |  |
| ERS_Oz_ | 0.093 | 0.009 | 0.895 | <0.001 |  |  |  |  |
| AxD | - | - | - | - |  |  | -0.072 | 0.449 |
| Right UF^b^ |  |  |  |  |  |  |  |  |
| Model 1 |  |  |  |  | 0.797 |  |  |  |
| ERS_Oz_ | 0.094 | 0.009 | 0.897 | <0.001 |  |  |  |  |
| FA | - | - | - | - |  |  | 0.166 | 0.075 |
| Model 2 |  |  |  |  | 0.797 |  |  |  |
| ERS_Oz_ | 0.094 | 0.009 | 0.897 | <0.001 |  |  |  |  |
| MD | - | - | - | - |  |  | -0.135 | 0.142 |
| Model 3 |  |  |  |  | 0.797 |  |  |  |
| ERS_Oz_ | 0.094 | 0.009 | 0.897 | <0.001 |  |  |  |  |
| RD | - | - | - | - |  |  | -0.154 | 0.095 |
| Model 4 |  |  |  |  | 0.797 |  |  |  |
| ERS_Oz_ | 0.094 | 0.009 | 0.897 | <0.001 |  |  |  |  |
| AxD | - | - | - | - |  |  | -0.098 | 0.289 |
| Left VOF^c^ |  |  |  |  |  |  |  |  |
| Model 1 |  |  |  |  | 0.792 |  |  |  |
| ERS_Oz_ | 0.093 | 0.009 | 0.895 | <0.001 |  |  |  |  |
| FA | - | - | - | - |  |  | -0.002 | 0.984 |
| Model 2 |  |  |  |  | 0.792 |  |  |  |
| ERS_Oz_ | 0.093 | 0.009 | 0.895 | <0.001 |  |  |  |  |
| MD | - | - | - | - |  |  | -0.127 | 0.172 |
| Model 3 |  |  |  |  | 0.792 |  |  |  |
| ERS_Oz_ | 0.093 | 0.009 | 0.895 | <0.001 |  |  |  |  |
| RD | - | - | - | - |  |  | -0.101 | 0.276 |
| Model 4 |  |  |  |  | 0.792 |  |  |  |
| ERS_Oz_ | 0.093 | 0.009 | 0.895 | <0.001 |  |  |  |  |
| AxD | - | - | - | - |  |  | -0.128 | 0.175 |
| Right VOF^d^ |  |  |  |  |  |  |  |  |
| Model 1 |  |  |  |  | 0.797 |  |  |  |
| ERS_Oz_ | 0.094 | 0.009 | 0.897 | <0.001 |  |  |  |  |
| FA | - | - | - | - |  |  | 0.050 | 0.587 |
| Model 2 |  |  |  |  | 0.797 |  |  |  |
| ERS_Oz_ | 0.094 | 0.009 | 0.897 | <0.001 |  |  |  |  |
| MD | - | - | - | - |  |  | -0.172 | 0.065 |
| Model 3 |  |  |  |  | 0.797 |  |  |  |
| ERS_Oz_ | 0.094 | 0.009 | 0.897 | <0.001 |  |  |  |  |
| RD | - | - | - | - |  |  | -0.165 | 0.068 |
| Model 4 |  |  |  |  | 0.797 |  |  |  |
| ERS_Oz_ | 0.094 | 0.009 | 0.897 | <0.001 |  |  |  |  |
| AxD | - | - | - | - |  |  | -0.171 | 0.065 |

AxD=axial diffusivity; ERS_Oz_=event-related synchronization of gamma-rhythms at Oz; FA=fractional anisotropy; MD=mean diffusivity; SE=standard error; RD=radial diffusivity; UF= uncinate fasciculus; VOF=vertical occipital fasciculus;

^a^Forward stepwise multiple linear regression analyses computing the spectral Granger causality of gamma-rhythm connectivity from left occipital to left frontal region as a dependent variable and ERS_Oz_ and one of FA, MD, RD or AxD of left UF as independent variables

^b^Forward stepwise multiple linear regression analyses computing the spectral Granger causality of gamma-rhythm connectivity from right occipital to right frontal region as a dependent variable and ERS_Oz_ and one of FA, MD, RD or AxD of right UF as independent variables

^c^Forward stepwise multiple linear regression analyses computing the spectral Granger causality of gamma-rhythm connectivity from left occipital to left frontal region as a dependent variable and ERS_Oz_ and one of FA, MD, RD or Ax) of left VOF as independent variables

^d^Forward stepwise multiple linear regression analyses computing the spectral Granger causality of gamma-rhythm connectivity from right occipital to right frontal region as a dependent variable and ERS_Oz_ and one of FA, MD, RD or AxD of right VOF as independent variables

**Supplementary figure 1.**


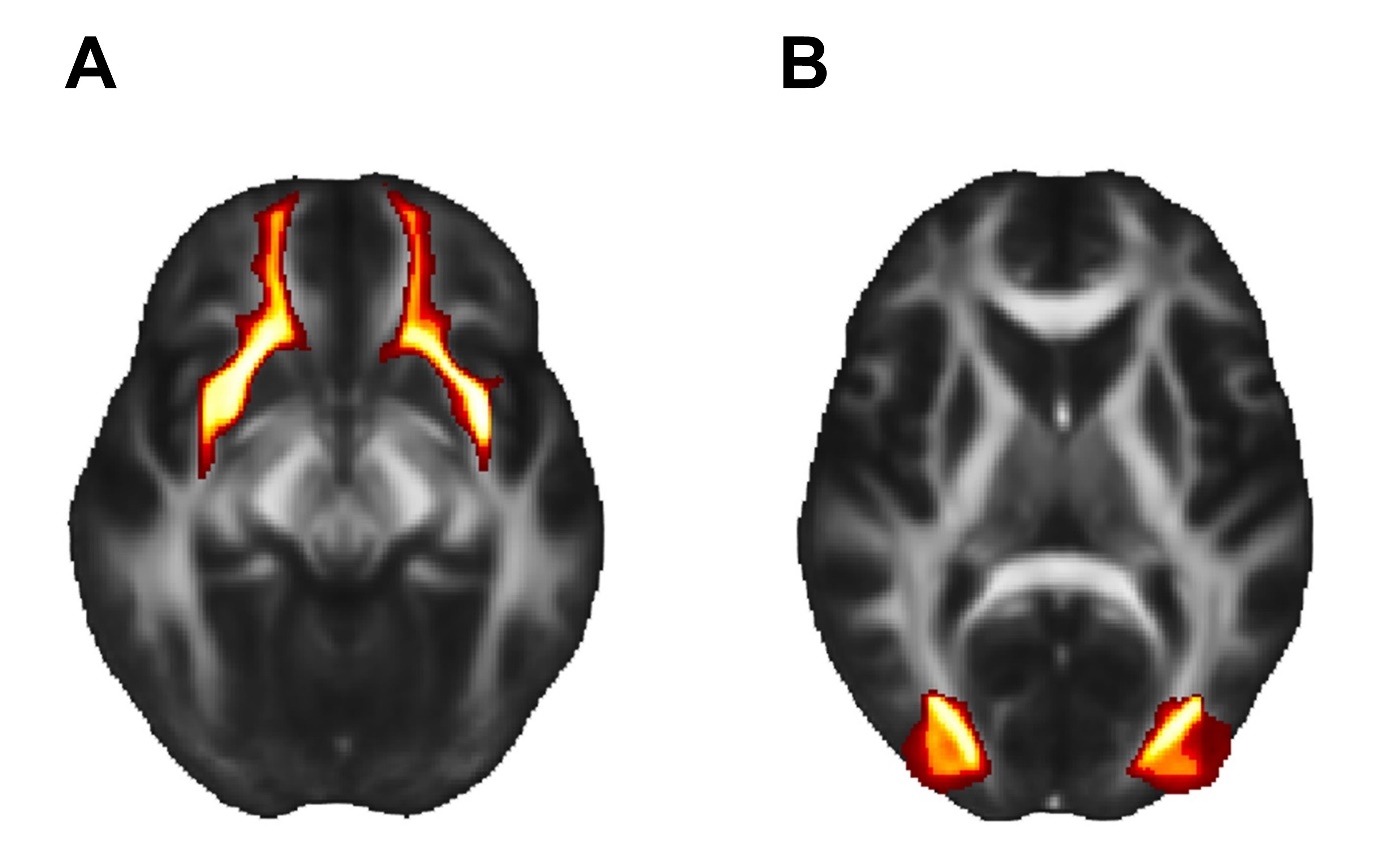


(A)Anatomical location of the uncinate fasciculus

(B)Anatomical location of the vertical occipital fasciculus
